# Effects of Shengjiang (*Zingiberis Rhizoma Recens*) and Its Processed Products on Nitric Oxide Production in Macrophage RAW 264.7 Cells

**DOI:** 10.1155/2015/828156

**Published:** 2015-03-18

**Authors:** Hui Liao

**Affiliations:** Department of Pharmacy, Shanxi Provincial People's Hospital, 29 Shuangtasi Street, Taiyuan, Shanxi 030012, China

## Abstract

In Chinese medicine, Shengjiang (*Zingiberis Rhizoma Recens*) and its processed products, such as Ganjiang (*Zingiberis Rhizoma*), Paojiang (*Zingiberis Rhizoma Preparatum*), and Jiangtan (*Zingiberis Rhizoma Carbonisata*), exert distinct efficacy clinically. This research tried to study the effects of extracts from Shengjiang and its processed products in RAW 264.7 macrophage cells. After incubation of the different ginger types in RAW 264.7 cells for 24 h, an aliquot of the culture was mixed with an equal volume of Griess reagent, and nitric oxide (NO) production was evaluated using a Griess assay. Lipopolysaccharide (LPS) was used as the positive control. Milli-Q water (MQW) was used as the solvent control. The results showed that NO production increased significantly in RAW 264.7 cells following the stimulation of LPS (0.05 *μ*g mL^−1^), Shengjiang, Ganjiang, Paojiang, and Jiangtan (50 *μ*g mL^−1^, 500 *μ*g mL^−1^) separately compared with the MQW control (*P* < 0.01). The stimulation effects of Shengjiang and Ganjiang were significantly higher than those of Paojiang and Jiangtan at different concentrations (*P* < 0.01). The conclusion we could get from this research is that Shengjiang and its processed products could induce NO production in RAW 264.7 cells.

## 1. Introduction

According to the Traditional Chinese Medicine theory, Shengjiang (*Zingiberis Rhizoma Recens*), the fresh rhizome of the Zingiberaceae plant ginger (*Zingiber officinale Rosc*), and its processed products, such as Ganjiang (*Zingiberis Rhizoma*), Paojiang (*Zingiberis Rhizoma Preparatum*), and Jiangtan (*Zingiberis Rhizoma Carbonisata*), can be used as medicines, and each of them exerts a distinct clinical efficacy [1, 2].

Modern studies have demonstrated that Ganjiang and Paojiang, the processed products of Shengjiang, both can inhibit the proliferation of lung cancer A549 cells and gastric cancer SGC-7901 cells [3]. A high concentration of nitric oxide (NO), as an important signaling molecule in organisms, can inhibit the proliferation of tumor cells. A large amount of NO generated after induction of the inducible nitric oxide synthase (iNOS) can cause death of tumor cells and inhibit their growth [4].

Studies have confirmed that stimulating factors such as lipopolysaccharide (LPS), interleukin-1, and the tumor necrosis factor can induce macrophages to highly express iNOS, thereby synthesizing NO [5]. It was found in our previous studies that Shengjiang had an effect of enhancing the generation of NO by LPS-induced mouse macrophage RAW 264.7 cells [6]. Moreover, it has been reported that Shengjiang* per se* had an effect of inducing RAW 264.7 cells to produce NO [7]. This study will further explore whether the aqueous extracts of Shengjiang and its processed products, that is, Ganjiang, Paojiang, and Jiangtan, can induce RAW 264.7 cells to generate NO and what impact the extracts of Shengjiang, Ganjiang, Paojiang, and Jiangtan have on the generation of NO by LPS-induced RAW 264.7 cells.

## 2. Methods

### 2.1. Preparation of Shengjiang and Its Processed Products

Shengjiang (*Zingiberis Rhizoma Recens*): the fresh rhizome of the Zingiberaceae plant ginger (*Zingiber officinale Rosc*) was treated by removing fibrous roots, mud, and sand, washed clean, and cut into thick slices upon use. Ganjiang (*Zingiberis Rhizoma*): the fresh rhizome of the plant ginger was treated by removing fibrous roots, mud, and sand, was washed clean, and was dried at a low temperature (40–45)°C. Paojiang (*Zingiberis Rhizoma Preparatum*): the Ganjiang was heated with sand until it was plumped up and turned brown at the surface and yellow inside; then, it was sprayed with a small amount of clean water, removed, and dried in the sun. Jiangtan (*Zingiberis Rhizoma Carbonisata*): the Ganjiang was carbonized by stir-frying until the surface turned black and the interior turned brown [1].

All the samples were identified by the Traditional Chinese Herb Division, Pharmacy Department of the Shanxi Provincial People's Hospital. The processing standards for each sample are shown in Table 1 [1].

Three grams of each sample was hermetically pulverized and then placed into a sealed tube. Then, 15 mL of Milli-Q water (MQW) at 100°C was added, sealed, shaken at room temperature for 1 h, and then ultrasonically extracted in a water bath at 60–65°C for 15 min. The extraction was repeated another two times same as above. The extracting solutions were collected, pooled, and centrifuged at 14,000 g for 15 min. The supernatant was removed and freeze-dried* in vacuo* to a constant weight to obtain extracts of Shengjiang, Ganjiang, Paojiang, and Jiangtan, respectively. The extracts were stored at 4°C until use. The extracts were redissolved in MQW at a desired concentration (50 *μ*g mL^−1^ and 500 *μ*g mL^−1^) upon experiment.

### 2.2. Reagents and Instruments

Lipopolysaccharide (LPS), sodium nitrite (NaNO_2_), and Griess reagent (0.1% N-(1-naphthyl)ethylenediamine dihydrochloride, 1% sulfanilamide in 5% phosphoric acid) were all prepared in-house from reagents purchased from Wako Chemicals USA Inc. (Richmond, VA, USA).

The used Victor2 plate reader (Wallac, Turku, Finland), 96-well polystyrene microplates, and Wallac Delfia 1296-003 shaker were purchased from PerkinElmer (Boston, MA, USA).

### 2.3. Cell Source and Culture

RAW 264.7 cells, a mouse macrophages cell line, were purchased from the American Type Culture Collection (Manassas, VA, USA) and cultured in colorless Dulbecco's modified eagle medium (DMEM) supplemented with heat inactivated fetal bovine serum (10%), D-glucose (3.5 mg mL^−1^), Na pyruvate (100 mM), L-glutamine (2 mM), penicillin (100 U mL^−1^), streptomycin (100 *μ*g mL^−1^), and amphotericin B (250 *μ*g mL^−1^) at 37°C and 5% CO_2_.

### 2.4. Effects of Shengjiang, Ganjiang, Paojiang, and Jiangtan in RAW 264.7 Cells

The effects of the extracts were determined in RAW 264.7 cells (99 *μ*L, plated at 1 × 10^6^ cells mL^−1^). The cells were treated with extracts (1 *μ*L). Nitrite, a stable end-product of NO metabolism, was then measured after 24 h using the Griess reaction [8]. The culture media of the RAW 264.7 cells (80 *μ*L) were mixed with an equal volume of Griess reagent, followed by spectrophotometric measurement at 550 nm. The nitrite concentrations in the culture media were determined by comparison with a NaNO_2_ standard curve. LPS (1 *μ*L, diluted with DMEM, 0.05 *μ*g mL^−1^) was the positive control, and MQW was the solvent control. Each concentration was assayed 6 times.

### 2.5. Effects of Shengjiang, Ganjiang, Paojiang, and Jiangtan in LPS-Activated RAW 264.7 Cells

The activities of inducible nitric oxide synthase (iNOS) of the extracts were determined with RAW 264.7 cells (98 *μ*L, plated at 1 × 10^6^ cells mL^−1^). The extracts (1 *μ*L) were added to the cells, and then, cells were stimulated with LPS (1 *μ*L, diluted with DMEM, 0.05 *μ*g mL^−1^) after 2 h. Nitrite was measured after another 22 h using the Griess reaction as above. Each concentration was assayed 6 times.

### 2.6. Statistics

All data are expressed as the mean ± standard deviation. Statistical analyses of the results were performed using SPSS software version12.0 (SPSS Inc., Chicago, IL, USA). Student's *t*-test was used for intergroup comparison. *P* < 0.05 was considered statistically significant.

## 3. Results

### 3.1. Standard Equation of NaNO_2_

The concentrations of NaNO_2_ were 0, 1, 2, 5, 10, 50, 100, and 500 *μ*M. The standard equation of NaNO_2_ was as follows: *Y* (absorbance) = 0.0018 × (the concentration of NaNO_2_) + 0.0454, *R* ^2^ = 0.9994.

### 3.2. Effects of LPS in RAW 264.7 Cells

NO production was increased significantly by LPS stimulation compared with the MQW solvent control ((14.4 ± 2.2) *μ*M versus (0.8 ± 0.5) *μ*M, *P* < 0.001) (Table 2).

### 3.3. Effects of Shengjiang, Ganjiang, Paojiang, and Jiangtan in RAW 264.7 Cells

The extracts from Shengjiang, Ganjiang, Paojiang, and Jiangtan could stimulate NO production significantly as compared with the MQW solvent control at 50 *μ*g mL^−1^ and 500 *μ*g mL^−1^ (Jiangtan at 50 *μ*g mL^−1^ *P* = 0.008; others: *P* < 0.001). Shengjiang and Ganjiang displayed significant effects compared with Paojiang and Jiangtan at different concentrations (*P* < 0.001) (Table 2).

### 3.4. Effects of Shengjiang, Ganjiang, Paojiang, and Jiangtan in LPS-Activated RAW 264.7 Cells

**Figure 1:**
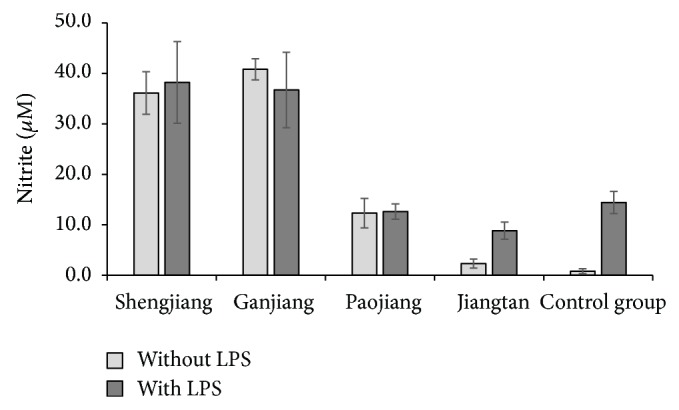
The effects of Shengjiang (*Zingiberis Rhizoma Recens*) and its processed herbs in RAW 264.7 cells at 50 *µ*g mL^−1^. LPS control compared with solvent control ((14.4 ± 2.2) *μ*M versus (0.8 ± 0.5) *μ*M, *P* < 0.001). “Jiangtan with LPS” compared with “Jiangtan without LPS” ((8.8 ± 1.7) *μ*M versus (2.3 ± 0.9) *μ*M, *P* < 0.001) and “Jiangtan with LPS” compared with “LPS control” ((8.8 ± 1.7) *μ*M versus (14.4 ± 2.2) *μ*M, *P* < 0.001).

**Figure 2:**
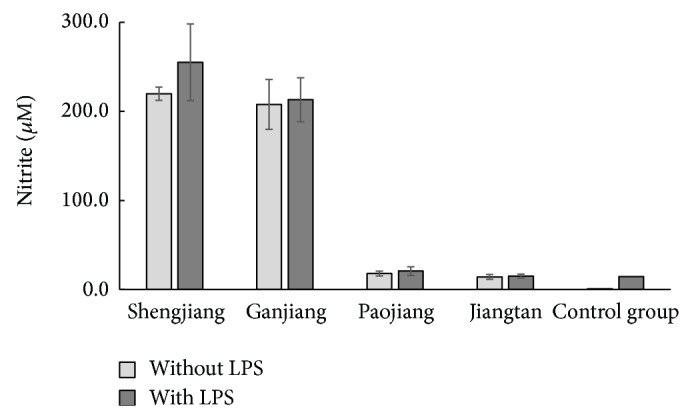
The effects of Shengjiang (*Zingiberis Rhizoma Recens*) and its processed herbs in RAW 264.7 cells at 500 *µ*g mL^−1^. “Paojiang with LPS” compared with “Paojiang without LPS” ((20.7 ± 4.8) *μ*M versus (17.9 ± 2.7) *μ*M, *P* = 0.029). “Paojiang with LPS” compared with “LPS control” ((20.7 ± 4.8) *μ*M versus (14.4 ± 2.2) *μ*M, *P* = 0.014).

When Shengjiang and Ganjiang at 50 *μ*g mL^−1^ and 500 *μ*g mL^−1^, Paojiang at 50 *μ*g mL^−1^, and Jiangtan at 500 *μ*g mL^−1^ were coadministered with LPS, no addition or synergy effects were observed (without LPS versus with LPS, resp., *P* > 0.05) (Figures 1 and 2).

When Paojiang at 500 *μ*g mL^−1^ was coadministered with LPS, the content of NO production was significantly higher than the test group without LPS ((20.7 ± 4.8) *μ*M versus (17.9 ± 2.7) *μ*M, *P* = 0.029) and also higher than LPS positive control significantly ((20.7 ± 4.8) *μ*M versus (14.4 ± 2.2) *μ*M, *P* = 0.014) (Figure 2).

When Jiangtan was coadministered with LPS at 50 *μ*g mL^−1^, the results showed that NO production was significantly higher than the test group without LPS ((8.8 ± 1.7) *μ*M versus (2.3 ± 0.9) *μ*M, *P* < 0.001), but lower than LPS control significantly ((8.8 ± 1.7) *μ*M versus (14.4 ± 2.2) *μ*M, *P* < 0.001) (Figure 1).

## 4. Discussion

Modern pharmacological studies have found that a high concentration of NO mainly has a cytotoxic effect and inhibits and kills tumor cells through mechanisms including mediating the activation of macrophages to achieve an antitumor effect. The results of the study in which chemotherapy with 5-fluorouracil (5-FU), together with supplementation of the NO precursor L-arginine (L-Arg), was administered to a nude mice model of human liver cancer revealed that 5-FU could induce an increase in the expression and activity of iNOS* in vivo*, and after combination with L-Arg 5-FU could significantly enhance the expression and activity of iNOS and significantly increase the concentration of NO in tumor tissues, suggesting that endogenous NO plays an important role in the* in vivo *antitumor effect of 5-FU combined with L-Arg [9].

Research data indicates that transfection of the iNOS gene into highly metastatic mouse malignant melanoma cells results in the high expression of iNOS activity, which causes a loss of tumor metastasis. Moreover, clinical data has confirmed that the concentration of NO in the serum of the patients with large intestine cancer was significantly increased after supplementation with L-Arg, indicating that the L-Arg-NO pathway is an action mechanism by which L-Arg inhibits tumor growth [10].

NO, which has a short half-life of 6 to 60 seconds, can be oxidized to nitrite immediately after its generation and exists in intracellular and extracellular fluids in the form of nitrite. Thus, in experiments, the content of nitrite, a stable product of NO, is usually measured to reflect the amount of NO [8]. At present, the model has been widely used in experimental studies on NO.

RAW 264.7 macrophages cell lines are thought to be very sensitive to the induction of mRNA expression by various cytokines via LPS stimulation [11]. In macrophages, macrophage-inducible NO synthase is mainly responsible for NO production in response to various stimuli [12]. Reverse transcription-polymerase chain reaction (RT-PCR) and Western Blotting were used to determine the change in iNOS after the stimulation of RAW 264.7 cells with LPS. The results demonstrated that, after 24-hour stimulation with 0.1 *μ*g mL^−1^ LPS, the levels of iNOS mRNA and protein expression in the cells were significantly increased and exhibited a dose-effect relationship within a concentration range of 0.01–10 *μ*g mL^−1^. It was considered that LPS could induce RAW 264.7 cells to produce iNOS, thereby increasing the NO content [13].

Our previous study showed that extracts from Shengjiang seemed to stimulate the NO production at 500 *μ*g mL^−1^ but had no effect at 50 *μ*g mL^−1^ [6]. It was interesting to see in another report that 100 *μ*g mL^−1^ extracts from Shengjiang could induce macNOS mRNA expression, but induction effects at a dose below 10 *μ*g mL^−1^ were weak or negligible [7]. In our study, Shengjiang was mixed with water and sonicated at room temperature [6], but samples were extracted by boiling in distilled water in another research [7]. Some research has demonstrated that Yanshanjiang (*Alpinia zerumbet*), another herb of the Zingiberaceae plant, could promote NO secretion. The active ingredient was its volatile oil [14]. It has been reported that some volatile oils in Ganjiang are significantly decreased when the drying temperature was 65°C [15]. It seemed that the extraction temperature is an important factor in the research. In this study, we extracted samples ultrasonically in a water bath at 60–65°C in sealed tubes and tried the concentrations of Shengjiang and its processed herbs at 500 *μ*g mL^−1^ and 50 *μ*g mL^−1^.

The results of this study indicate that the content of nitrite in RAW 264.7 cell culture after LPS induction was significantly increased from 0.8 ± 0.5 *μ*M to 14.4 ± 2.2 *μ*M, as compared with the solvent control group. At the action concentration of 500 *μ*g mL^−1^ and 50 *μ*g mL^−1^, the content of nitrite in the cell culture of each experimental group of Shengjiang, Ganjiang, Paojiang, and Jiangtan was significantly increased as compared with the solvent control group.

At the same concentration, the stimulation effects of Shengjiang and Ganjiang to produce nitrite, a product of NO, were significantly higher than those of Paojiang and Jiangtan. The main chemical components in Shengjiang are volatile oils and phenols, such as gingerol and shogaol [16]. The processing temperature plays an important role in determining the content of volatile oils in processed herbs. The high processing temperature might be responsible for the inducible effects of Paojiang and Jiangtan being significantly lower than Ganjiang and Shengjiang in this study.

When the doses of Shengjiang and Ganjiang were 500 *μ*g mL^−1^ and 50 *μ*g mL^−1^, the nitrite levels were significantly higher than that of the positive LPS control. Under the action of 500 *μ*g mL^−1^ of Shengjiang and Ganjiang, the nitrite content levels were 15 and 14 times as high as that of LPS, respectively. Most likely because the effects of Shengjiang and Ganjiang were significantly much higher than that of LPS, when Shengjiang and Ganjiang were coadministered with LPS, no additional or synergistic effect was demonstrated.

Even though the inducible effect of Shengjiang and Ganjiang did not display significant differences at the same concentration, Shengjiang and Ganjiang possessed both similarities and obvious differences in drug properties and efficacies and also had significant differences in clinical applications. There were significant differences in chromatographic peaks on their UPLC fingerprint chromatograms before and after processing [17], demonstrating that the ingredients in Shengjiang were “qualitatively” and “quantitatively” changed after the processing. It has been reported that over 60 compounds have been extracted from Ganjiang, and 40 of those compounds have been identified. In addition, over 50 compounds from Shengjiang have been extracted, and 33 of those compounds have been identified. There are 10 compounds in Ganjiang that Shengjiang does not have, and there are 3 compounds in Shengjiang that have not been detected in Ganjiang [18].

When Paojiang at 500 *μ*g mL^−1^ was coadministered with LPS, the content of NO production was significantly higher than single Paojiang, and also single LPS. It seemed there had been the synergistic effect of Paojiang at 500 *μ*g mL^−1^ and LPS in RAW 264.7 cells. Another interesting result was when Jiangtan was coadministered with LPS at 50 *μ*g mL^−1^. The data showed that NO production was significantly higher than single Jiangtan, but lower than single LPS. We are not sure that there is some inhibition effect after their combination and need more analytical research.

Accordingly, our further studies will focus on which ingredients play the role in the significant higher levels of nitrite treated with Shengjiang and Ganjiang compared to positive LPS control in RAW 264.7 cells. We are also interested in Paojiang and Jiangtan: How did the main active component change after high processed temperature? Will they have different pharmacological effect on RAW 264.7 cells compared with Shengjiang and Ganjiang? We will give further research on these questions.

## Figures and Tables

**Table 1:** Standard assay requirements for Shengjiang (*Zingiberis Rhizoma Recens*) and its processed herbs [1].

Different ginger types	Water content	Total ash	Water soluble extractive	Volatile oil	6-Gingerol
Shengjiang (*Zingiberis Rhizoma Recens*)		<2.0%			>0.050%
Ganjiang (*Zingiberis Rhizoma*)	<19.0%	<6.0%	>22%	>0.80%	>0.60%
Paojiang (*Zingiberis Rhizoma Praeparatum*)	<12.0%	<7.0%	>26%		>0.30%
Jiangtan (*Zingiberis Rhizoma Carbonisata*)			<26%		>0.050%

**Table 2:** Effects of Shengjiang (*Zingiberis Rhizoma Recens*) and its processed herbs in RAW 264.7 cells ($\overset{-}{x}\pm s$).

Group	Concentration (*μ*g mL^−1^)	Nitrite (*μ*M)
Shengjiang (*Zingiberis Rhizoma Recens*)	50	36.1 ± 4.2^abd^
	500	219.8 ± 7.3^ace^
Ganjiang (*Zingiberis Rhizoma*)	50	40.8 ± 2.1^abd^
	500	207.7 ± 28.0^ace^
Paojiang (*Zingiberis Rhizoma Praeparatum*)	50	12.3 ± 2.9^a^
	500	17.9 ± 2.7^a^
Jiangtan (*Zingiberis Rhizoma Carbonisata*)	50	2.3 ± 0.9^a^
	500	14.1 ± 2.7^a^
Solvent control		0.8 ± 0.5
LPS positive control	0.05	14.4 ± 2.2^a^

Notes: ^a^ *P* < 0.01, compared with MQW solvent control; ^b^ *P* < 0.01, Shengjiang and Ganjiang compared with Paojiang at 50 *μ*g mL^−1^; ^c^ *P* < 0.01, Shengjiang and Ganjiang compared with Paojiang at 500 *μ*g mL^−1^; ^d^ *P* < 0.01, Shengjiang and Ganjiang compared with Jiangtan at 50 *μ*g mL^−1^; ^e^ *P* < 0.01, Shengjiang and Ganjiang compared with Jiangtan at 500 *μ*g mL^−1^.
